# Subtotal nephrectomy inhibits the gastric emptying of liquid in awake rats

**DOI:** 10.14814/phy2.12291

**Published:** 2015-02-12

**Authors:** José Ronaldo Vasconcelos da Graça, Cynara Carvalho Parente, Robério Ferreira Fiúza, Pedro Alberto Freitas da Silva, Bruno Teixeira Mota, Luiz Derwal Salles, Camila Meirelles de Souza Silva, Moisés Tolentino Bento da Silva, Ricardo Brandt de Oliveira, Armenio Aguiar dos Santos

**Affiliations:** 1 School of Medicine, Federal University of CearáSobral and Fortaleza, Brazil; 2 Department of Physical Education, Federal University of PiauíTeresina, Brazil; 3 Department of Clinical Medicine, Ribeirão Preto School of Medicine, São Paulo UniversityRibeirão Preto, Brazil

**Keywords:** 5/6 Partial nephrectomy, azotemia, gastrointestinal motility, hypervolemia, intestinal transit

## Abstract

Homeostasis of blood volume (BV) is attained through a functional interaction between the cardiovascular and renal systems. The gastrointestinal tract also adjusts its permeability and motor behavior after acute BV imbalances. We evaluated the effect of progressive nephron loss on gut motility. Male Wistar rats were subjected or not (sham) to 5/6 partial nephrectomy (PNX) in two steps (0 and 7th day). After further 3, 7, or 14 days, PNX and sham operation (control) rats were instrumented to monitor mean arterial pressure (MAP), central venous pressure (CVP), heart rate (HR), and blood collection for biochemical analysis. The next day, they were gavage fed with a liquid test meal (phenol red in glucose solution), and fractional dye recovery determined 10, 20, or 30 min later. The effect of nonhypotensive hypovolemia and the role of neuroautonomic pathways on PNX-induced gastric emptying (GE) delay were also evaluated. Compared with the sham-operated group, PNX rats exhibited higher (*P *<* *0.05) MAP and CVP values as well as increased values of gastric dye recovery, phenomenon proportional to the BV values. Gastric retention was prevented by prior hypovolemia, bilateral subdiaphragmatic vagotomy, coelic ganglionectomy + splanchnicectomy, guanethidine, or atropine pretreatment. PNX also inhibited (*P *<* *0.05) the marker's progression through the small intestine. In anesthetized rats, PNX increased (*P *<* *0.05) gastric volume, measured by a balloon catheter in a barostat system. In conclusion, the progressive loss of kidney function delayed the GE rate, which may contribute to gut dysmotility complaints associated with severe renal failure.

## Introduction

Since the advent of hemodialysis (HD) 50 years ago, the natural history of chronic renal disease has changed dramatically, prolonging the survival of patients with end-stage renal disease (ESRD). Although the vast majority of ESRD patients suffer from complications of diabetes mellitus and systemic arterial hypertension, their most common, chronic, nonrenal problem is related to gut complaints, including dyspepsia, nausea, vomiting, abdominal pain, bloating, heartburn, constipation, and diarrhea (Van Vlem et al. 2001; Strid et al. 2004).

However, the impact of gut dysmotility in ESRD remains still unclear. Some authors have reported that patients on regular HD therapy have normal rates of gastric emptying (GE) of solid and liquid meals (Soffer et al. 1987; Hirata et al. 2012), whereas others reported a delay of GE of solids (Van Vlem et al. 2000) or a normal rate of GE of solids but speedy GE of liquids (Dumitrascu et al. 1975). In fact, peritoneal dialysate may delay the GE of a solid test meal (Brown-Cartwright et al. 1988). An explanation for this apparent paradox is that such clinical trials included subjects with important differences in age, gender, the duration of renal failure, the extent of nephron lesion, and the dialysis regimen.

We showed that bilateral nephrectomy delays the GE of a liquid test meal in awake rats, a phenomenon that was present as early as 6 h after kidney exeresis and directly proportional to the increase in blood volume (BV) (Silva et al. 2002). Although these results provided a new approach for analysis of such phenomenon, important questions remained. Bilateral nephrectomy concentrates several nitrogen compounds in the extracellular fluid (ECF) space that may inhibit the contractility of gut smooth muscle (Lin et al. 1997). Thus, we reassessed this issue using a remnant kidney model that mimics the progressive nephron loss associated with ESRD (Fleck et al. 2006). We also investigated the role of neuroautonomic pathways in this condition.

## Material and Methods

All of the studies were performed in accordance with the *Ethical Principles for Care and Use of Laboratory Animals* by the Brazilian Society for Laboratory Animal Science after approval from the local ethics committee (protocol no. 46/07). Male Wistar rats (230–280 g, *n *=* *138) were obtained from the central housing facility of the Federal University of Ceará and maintained in a temperature-controlled room on a 12 h/12 h light/dark cycle.

### Surgical procedures

After anesthesia (10 mg kg^−1^ xylazine and 25 mg kg^−1^ ketamine, i.m.), the rats were randomly subjected to right lumbar incision and dissection of the renal pedicle, followed or not (sham surgery) by subtotal nephrectomy according to the protocol described by Amann et al. (1993). In the subtotal nephrectomy group, the manipulation was followed by organ removal. One week later, the rats were anesthetized again. After a left lumbar incision was made, the poles of the left kidney were resected, excising 5/6 of the total renal parenchyma. Hemostasis of the kidney stump was attained using an absorbable sponge (Surgicel, Ethicon, Johnson & Johnson, New Brunswick, NJ). The control rats were subjected to a two-stage surgical procedure, also consisting of lumbar incision and renal manipulation, but both kidneys remained intact in the abdominal cavity. Surgical care was taken to avoid lesion of the adrenals.

One week after the first surgery (left lumbar incision), the rats were anesthetized again for the insertion of polyethylene tubes into the right femoral vessels (artery and vein) and jugular vein. The distal ends of the catheters were subcutaneously channeled, exteriorized, and fixed by a silk suture at the interscapular region. Additionally, three stainless-steel, Teflon-coated wires (0.203 mm outer diameter, A.M. Systems, Carlsborg, WA) were affixed to the chest muscles and hip muscle of the left paw and then exteriorized and fixed. After the wires were connected to a bioamplifier (ML132 BioAmp) coupled to a data acquisition system (PowerLab/8SP, AD Instruments, Bella Vista, Australia), an electrocardiographic signal could be derived allowing continuous recording of heart rate (HR; in beats min^−1^). Connection of the arterial and venous catheters to pressure transducers coupled to the data acquisition system allowed the continuous monitoring of mean arterial pressure (MAP; in mmHg) and central venous pressure (CVP; in cmH_2_O), respectively.

A day before the studies, the rats were kept isolated in metabolic cages and fasted for 18 h with free access to an oral rehydration solution (ORS; 75 mmol/L Na^+^, 65 mmol/L Cl^−^, 20 mmol/L K^+^, 75 mmol/L glucose, and 10 mmol/L citrate). This allowed the rats to clear their stomachs of residue while maintaining euglycemia (Souza et al. 2009). At the time of study, the rats were subjected to a 50-min period of continuous hemodynamic monitoring. The first 20-min period was considered the *basal interval*.

### Gastrointestinal motility assessments

After *3*, *7*, or *14* days of the 5/6 partial nephrectomy (PNX) or sham protocol, we used a dye dilution technique, originally described by Reynell and Spray (1956) and adapted in our laboratory (Souza et al. 2009), for the GE and intestinal transit measurements. After the basal interval, the rats were gavage fed with a liquid test meal (1.5 mL) that contained a nonabsorbable marker (0.5 mg mL^−1^ of phenol red in 5% glucose solution). After 10, 20, or 30 min, the rats were sacrificed by cervical dislocation. The gut was quickly exposed by laparotomy, clamped at the pylorus, cardia, and ileo-cecal junction, and then removed. The gut was carefully stretched from the stomach to colon along a meter-stick on a plain tabletop and divided into four consecutive segments: stomach, proximal small intestine (first 40%), middle small intestine (intermediate 30%), and distal small intestine (last 30%). Each segment was placed into a graduated cylinder, and the volume was measured by adding 100 mL of 0.1 N NaOH. After being cut into small pieces and homogenized for 30 s, the suspension was allowed to settle for 20 min at room temperature. The supernatant (10 mL) was then centrifuged for 10 min at 2800 rotations per minute (rpm), and the proteins in 5 mL of the homogenate were precipitated by 0.5 mL trichloroacetic acid (20% w/v). After additional centrifugation for 20 min at 2800 rpm, 3 mL of the supernatant was added to 4 mL of 0.5 N NaOH. A standard dilution curve was generated in each study that related the concentration of phenol red in 0.1 N NaOH to absorbance at 560 nm. A linear coefficient of the standard dilution curve (*α*) was used to define the concentration of the sample read and amount of dye recovered from each segment. Gastric fractional dye recovery was determined according to the following equation: *% gastric recovery = amount of phenol red recovered in the stomach / total amount of phenol red recovered from all four segments × 100*.

To evaluate the role of BV on the present phenomenon, we subjected a separate group of rats to identical sham operation or 5/6 partial nephrectomy (PNX). One day later, they received a bilateral dorsal injection (2.5 mL, s.c.) of polyethylene glycol (MW 20,000) solution (30%). After 4 h, the rats were subjected to basal hemodynamic monitoring, gavage fed with the test meal, and sacrificed 20 min later for gastric dye recovery analysis, as described above (Gondim et al. 1999). As we previously reported (Palheta et al. 2013), such subcutaneous injection of the colloidal solution elicits a large sequestration of extracellular fluid at the injection site.

To evaluate the role of the autonomic nervous system on the present phenomenon, a separate group of sham or PNX rats was anesthetized (10 mg kg^−1^ xylazine and 25 mg kg^−1^ ketamine, i.m.), and subjected or not to bilateral subdiaphragmatic vagotomy or celiac ganglionectomy + splanchnicectomy (Palheta-Junior et al. 2013). After 3 days, the rats were gavage fed with the test meal, and sacrificed 10 min later for gastric dye recovery analysis as described above. To elucidate the role of adrenergic and cholinergic pathways in the present phenomenon, we subjected a separate group of rats to identical sham or PNX protocol. One day later, they received intraperitoneal (i.p) injection (0.1 mL kg^−1^) of atropine (1 mg kg^−1^) or guanethidine (10 mg kg^−1^). They were then gavage fed with the test meal and sacrificed 10 min later for gastric dye recovery analysis, as described above.

### Small intestine transit assessment

To assess the effect of PNX on small intestine transit, another set of rats underwent a different surgery. After anesthesia with xylazine and ketamine (10 and 25 mg kg^−1^, respectively; i.m.), they were subjected to laparotomy to perform a small section of the gastric fundus into which a cannula was inserted into the gut. The tip of the cannula was advanced to the duodenum 1 cm distal to the pylorus. The cannula was fixed at the stomach wall by a purse-string suture, and its free end was subcutaneously channeled, exteriorized, and fixed to the skin. The rats were then subjected to identical sham operation or PNX protocol. One day later, we performed basal hemodynamic monitoring, followed by gavage feeding with the liquid test meal (1.0 mL) via the duodenal cannula. The animals were sacrificed 20 min later.

After gut exeresis, the stomach and first 1 cm of the duodenum that contained the cannula tip comprised segment 1. The remaining gut was carefully stretched and removed. Obstructive ligatures were performed to obtain five consecutive segments of the small intestine (approximately 20 cm long). Each segment was homogenized, and its dye content determined spectrophotometrically, as described above. Fractional dye recovery was calculated for each gut segment as the ratio between the mass obtained in it and the sum of the mass in all of the segments, including the gastroduodenal segment. The data obtained for each individual segment were multiplied by the number of respective segments and summed to calculate the geometric center of the marker distribution throughout the gut (Silva et al. 2014).

### Gastric tonus assessment

To verify the effect of PNX on gastric tonus, another group of rats was studied using a barostatic system. (Silva et al. 2014). Seven days after the sham or PNX procedure, separate groups of rats were anesthetized with urethane (1.2 g/kg ip) followed by tracheostomy. A balloon catheter (∼ 4 mL) made of surgical glove fingertips was then orally inserted and positioned in the rat's proximal stomach. Its free end was connected to a U-shaped glass reservoir (2.5 cm inner diameter, 30 mL volume), creating a communicant vessel system filled with an ionic standard solution (45 mg% NaCl and 0.3 mL% of Imbebient, BBC Ornano, Comerio, Italy), prewarmed at 37°C. Thus, the changes in reservoir volume, continuously displayed by a plethysmometer (model 7140, Ugo Basille, Comerio, Italy), could be taken as gastric volume. After system equilibration, the stomach was progressively distended by raising the reservoir liquid level 4.0, 8.0, and 12.0 cm above the rat's xyphoid appendix every 10 min. Gastric volume was recorded every 1 min and pooled into consecutive 10-min intervals, referred to as *4.0*, *8.0*, and *12.0*.

### Biochemical measurements

At the end of study, blood samples were collected by cardiac puncture and then centrifuged at 2800 rpm for 10 min. Serum urea and creatinine levels (in mg dL^−1^) were determined via standard colorimetric tests (Labtest, Labtest Diagnóstica S.A). Serum chloride, sodium, and potassium concentrations (in mEq L^−1^) were obtained by flame photometry (FC-180 CELM). Blood volume (in mL 100 g^−1^ body weight) was calculated from the plasma volume (PV) after arterial hematocrit (Hto) correction. The rats received an intravenous injection (1 mL) of Evans blue solution (0.5 mg mL^−1^). Following the dye injection, a curve of concentration change in time could be recorded downstream in the aorta. The calculation can be summarized by comparison with the direct Fick procedure for measurement of the cardiac output. In the present work, an arterial blood sample was collected 10 min later to determine Hto and PV according to the dye dilution technique (Ertl et al. 2007).

### Statistical analysis

The hemodynamic data were pooled into consecutive intervals and are presented as mean ± SEM. Repeated measures one-way analysis of variance (ANOVA), followed by Friedman's *post hoc* test, was used to compare differences in mean GE, CVP, MAP, and HR values between the basal and time intervals within and between groups. Values of *P *<* *0.05 were considered statistically significant.

## Results

**Figure 1:**
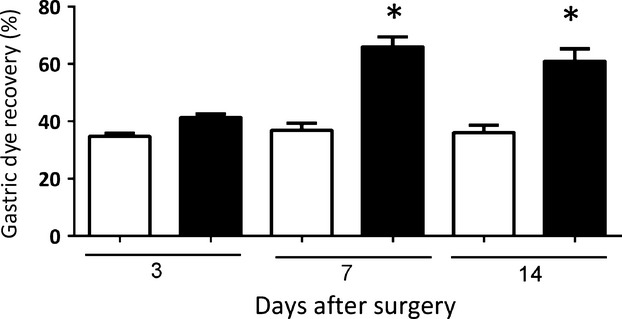
Effects of 5/6 nephrectomy (PNX, ▪) and sham nephrectomy (control, □) on the gastric retention of a liquid test meal in awake rats. Three, 7, or 14 days after surgery, the animals were gavage fed (1.5 mL) the test meal (0.5 mg mL^−1^ phenol red in 5% glucose solution) and sacrificed 20 min later. Gastric dye recovery was determined spectrophotometrically. Each control and experimental subgroup consisted of six to eight rats. The bars indicate mean gastric fractional dye recovery values, and the respective vertical lines denote the SEM. *, *P *<* *0.05 versus respective control rats after ANOVA and Student–Newman–Keuls test.

**Figure 2:**
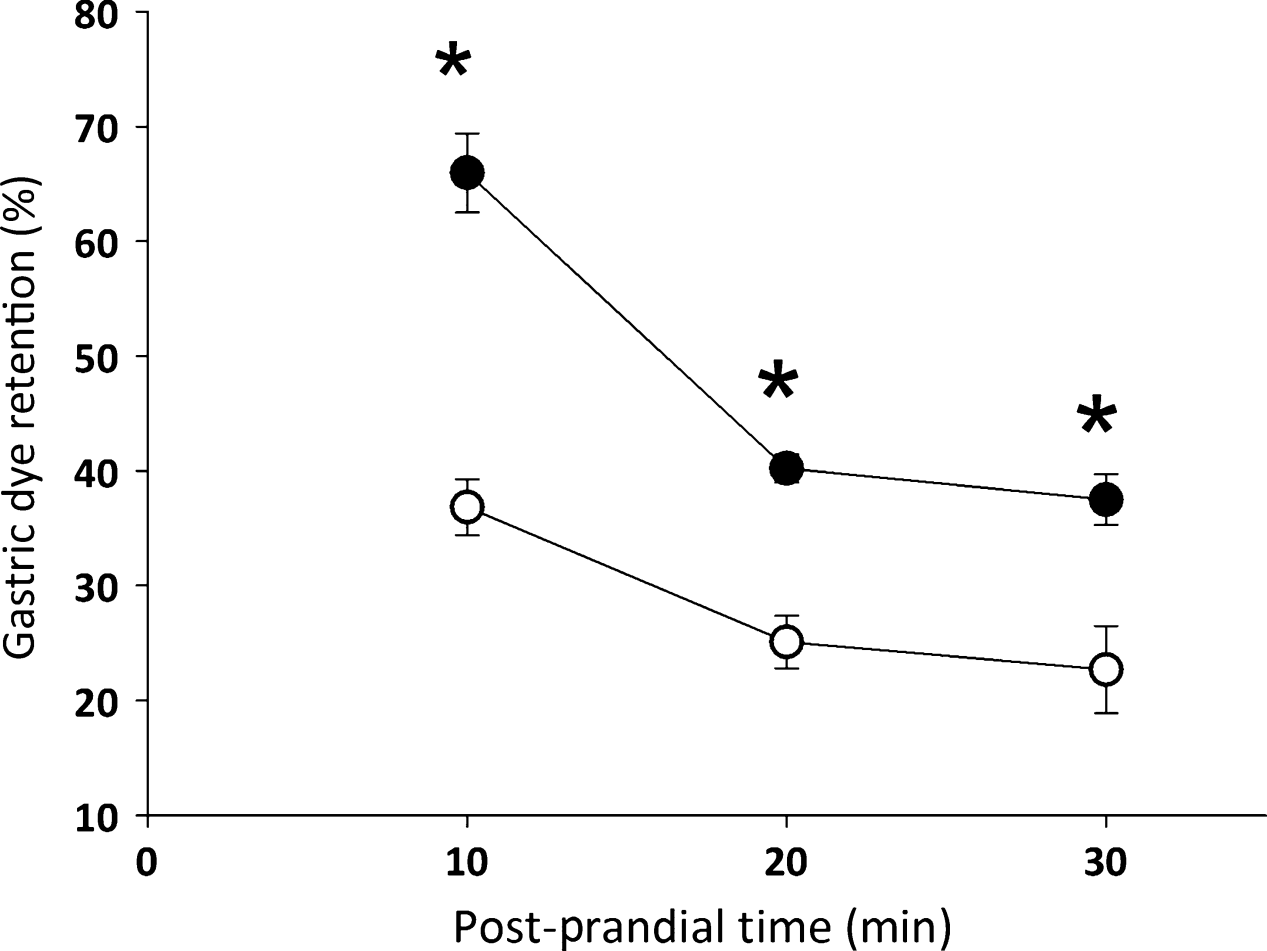
Effects of 5/6 nephrectomy (PNX, •) and sham nephrectomy (control, ○) on the gastric retention of a liquid test meal in awake rats. Seven days after surgery, the animals were gavage fed (1.5 mL) the test meal (0.5 mg mL^−1^ phenol red in 5% glucose solution) and sacrificed 10, 20, or 30 min later. Gastric dye recovery was determined spectrophotometrically. Each control and experimental subgroup consisted of six to eight rats. The data points indicate the mean fractional gastric recovery values, and the respective vertical lines denote the SEM. *P <* *0.05 versus respective postprandial interval in control rats after ANOVA and Student–Newman–Keuls test.

Figure1 shows that PNX increased gastric fractional dye retention of a liquid test meal compared with sham-operated rats. This effect was present in animals studied 7 or 14 days after surgery (36.8 ± 2.4 vs. 65.9 ± 3.4 and 36.0 ± 2.5% vs. 60.9 ± 4.3% in sham-operated and 5/6 partial nephrectomized rats, *P *<* *0.05, respectively), but it was not statistically significant in those studied 3 days after surgery (39.3 ± 5.5 vs. 43.8 ± 3.5% in sham-operated and 5/6 partial nephrectomized rats). Moreover, in animals studied 7 days after surgery, gastric fractional dye recovery at the 10, 20, and 30 min postprandial intervals was consistently higher (*P *<* *0.05) in the PNX group than in sham-operated one (65.9 ± 3.4 vs. 36.8 ± 2.4%, 38.4 ± 3.5 vs. 28.6 ± 3.3%, and 32.4 ± 3.6 vs. 21.3 ± 2.6%, respectively; Fig.2).

**Figure 3:**
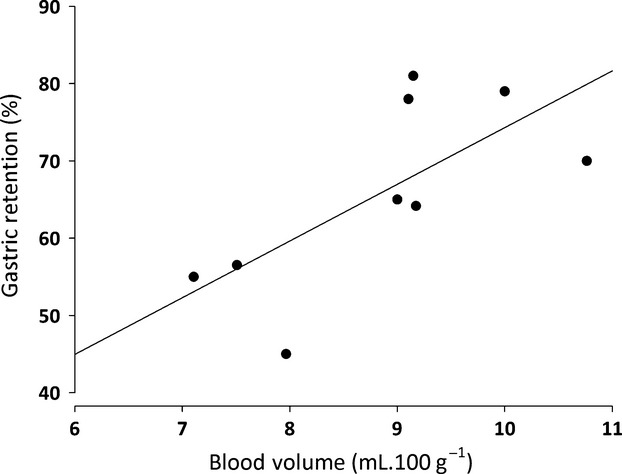
Correlation between gastric fractional dye retention of a liquid test meal and blood volume in awake rats previously (7 days) subjected to 5/6 nephrectomy (PNX). A strong correlation (*r*^2* *^= 0.86) was found between gastric retention and blood volume in PNX rats, indicated by the linear regression equation *y* = −2.8*x* + 75.4.

Table1 shows that PNX significantly increased (*P *<* *0.05) the BV from respective control levels in the sham-operated group (7.1 ± 0.1 vs. 8.9 ± 0.4 mL 100 g^−1^ body weight, respectively). Moreover, a strong (*r*^2* *^= 0.86) positive correlation was found between the increase in BV and increase in fractional dye recovery from the stomach of rats subjected to PNX, as indicated by a linear regression equation (*y* = -2.8*x* + 75.4; Fig.3).

**Table 1:** Hemodynamic and biochemical plasma parameters. Comparison of hemodynamic and plasma biochemical indices in awake sham-operated and 5/6 nephrectomized rats studied 7 days after surgery. After 18 h of fasting with free access to an oral rehydration solution, the rats were subjected to 20-min basal hemodynamic monitoring followed by gavage feeding of the liquid test meal. Mean arterial pressure (MAP, in mmHg) and central venous pressure (CVP, in cmH_2_O) were continuously recorded for 50 min using a digital setup. Heart rate (HR, in beats min^−1^) was derived from the electrocardiographic signal. The plasma volume values (in mL 100 g^−1^ body weight) were determined by the Evans blue method. The plasma concentrations of urea and creatinine (in mg dL^−1^) were determined by an automatic analyzer (Stat Profile Plus.9, Nova Medical Corp). The plasma Na^+^, K^+^, and Cl^-^ values (in mg dL^−1^) were determined by flame photometry. The data are expressed as mean ± SEM. The number of observations in each subgroup varied from 4 to 8.

	Sham	Nephrectomy
MAP (mmHg)	101.0 ± 2.4	121.8 ± 2.7*
CVP (cmH_2_O)	1.3 ± 0.4	2.6 ± 0.2*
HR (beats min^−1^)	387.2 ± 10.5	402.1 ± 15.5
Blood volume (mL 100 g^−1^)	7.1 ± 0.1	8.9 ± 0.4*
	Sham	Nephrectomy
Urea (mg dL^−1^)	45.8 ± 3.1	69.3 ± 2.5*
Creatinine (mg dL^−1^)	0.64 ± 0.05	0.96 ± 0.07*
Na^+^ (mEq L^−1^)	138.0 ± 1.7	141.1 ± 1.5
K^+^ (mEq L^−1^)	3.8 ± 0.1	7.1 ± 0.5
Cl^-^ (mEq L^−1^)	102.5 ± 1.2	99.2 ± 1.0

* *P *<* *0.05, versus respective sham-operated group (ANOVA and Student–Newman–Keuls test).

**Figure 4:**
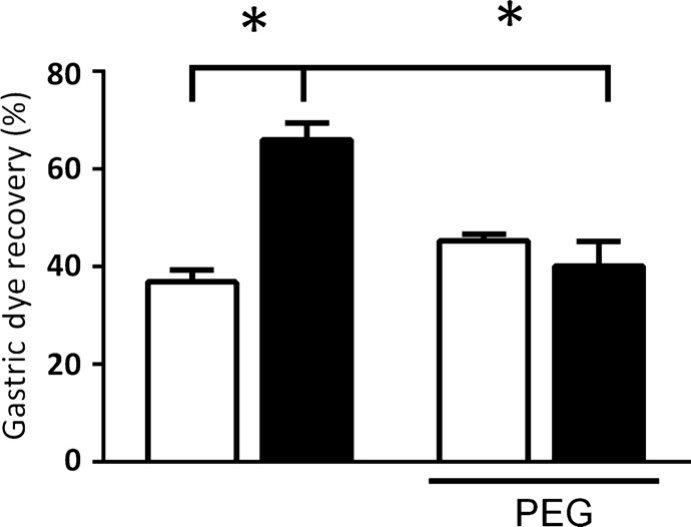
Effects of polyethylene glycol (PEG) treatment on 5/6 nephrectomy (PNX, ▪)-induced gastric emptying delay of liquid in awake rats. Seven days after nephron mass loss or sham operation (control, □), the rats were gavage fed (1.5 mL) the test meal (0.5 mg mL^−1^ phenol red in 5% glucose solution) and sacrificed 10 min later. Gastric dye recovery was determined spectrophotometrically. Rats that were subjected or not (control) 30 min earlier to polyethylene glycol (PEG) pretreatment. Each control and experimental subgroup consisted of six to eight rats. The data are expressed as mean ± SEM. *P <* *0.05, compared with respective sham-operated and sham-treated rats (ANOVA and Student–Newman–Keuls test.

The influence of hypervolemia on the present phenomenon is further emphasized in Figure4, showing that acute cellular dehydration caused by polyethylene glycol treatment prevented the PNX-induced GE delay in awake rats. No difference in gastric fractional dye recovery values was found between sham-operated and PNX rats that received polyethylene glycol (45.3 ± 1.3 vs. 40.0 ± 5.0%, respectively), although it lowered (*P *<* *0.05) *per se* the gastric fractional dye recovery from the respective levels in the PNX-vehicle-treated group (65.9 ± 3.4%).

**Figure 5:**
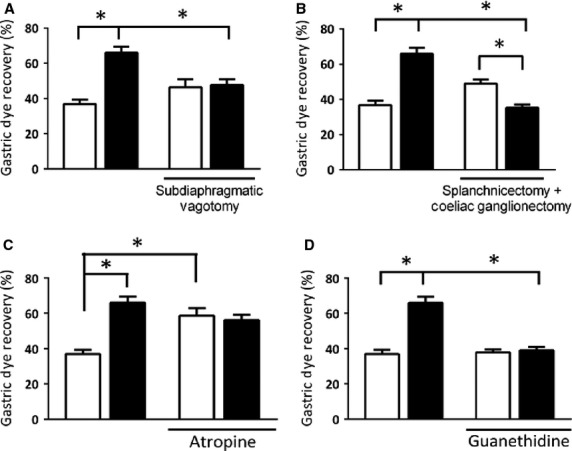
Effects of neuroautonomic extrinsic denervation, atropine, and guanethidine treatments on 5/6 nephrectomy (PNX, ▪)-induced gastric emptying delay of liquid in awake rats. Seven days after nephron mass loss or sham operation (control, □), the rats were gavage fed (1.5 mL) the test meal (0.5 mg mL^−1^ phenol red in 5% glucose solution) and sacrificed 20 min later. Gastric dye recovery was determined spectrophotometrically. (A) Rats that were subjected or not (control) 5 days earlier to bilateral subdiaphragmatic vagotomy. (B) Rats that were subjected or not (control) 5 days earlier to coeliac ganglionectomy + splanchnicectomy. (C) Rats that were subjected or not (control) 30 min earlier to atropine pretreatment. (D) Rats that were subjected or not (control) 30 min earlier guanethidine pretreatment. Each control and experimental subgroup consisted of six to eight rats. The data are expressed as mean ± SEM. *P <* *0.05, compared with respective sham-operated and sham-treated rats (ANOVA and Student-Newman-Keuls’ test).

The role of neuroautonomic pathways in the present phenomenon is also shown in Figure5, indicating that bilateral subdiaphragmatic vagotomy prevented the PNX-induced GE delay in awake rats (Fig.5A), but coelic ganglionectomy + splanchnicectomy did not (Fig.5B). The bilateral subdiaphragmatic vagotomy did not alter significantly *per se* the gastric fractional dye recovery of control (sham-operated) rats (36.8 ± 2.4%) and there is no difference between the gastric recovery values of vagotomized rats subjected or not to PNX (47.6 ± 3.2 vs. 46.4 ± 4.5%, respectively). By its turn, coelic ganglionectomy + splanchnicectomy reverted the PNX-induced GE delay (Fig.5B). As a matter of fact, the gastric fractional dye recovery values of PNX rats became significantly lower after coelic ganglionectomy + splanchnicectomy (49.0 ± 2.3 vs. 35.3 ± 1.9%). Moreover, the present phenomenon involved cholinergic and adrenergic pathways (Fig.5C and D) since there is no difference between the gastric fractional recovery values of atropinized rats subjected or not to PNX (56.1 ± 2.8 vs. 58.5 ± 4.1%, respectively) nor in guanethidine-treated rats previously subjected or not to PNX (38.9 ± 1.9 vs. 37.8 ± 4.1%).

**Figure 6:**
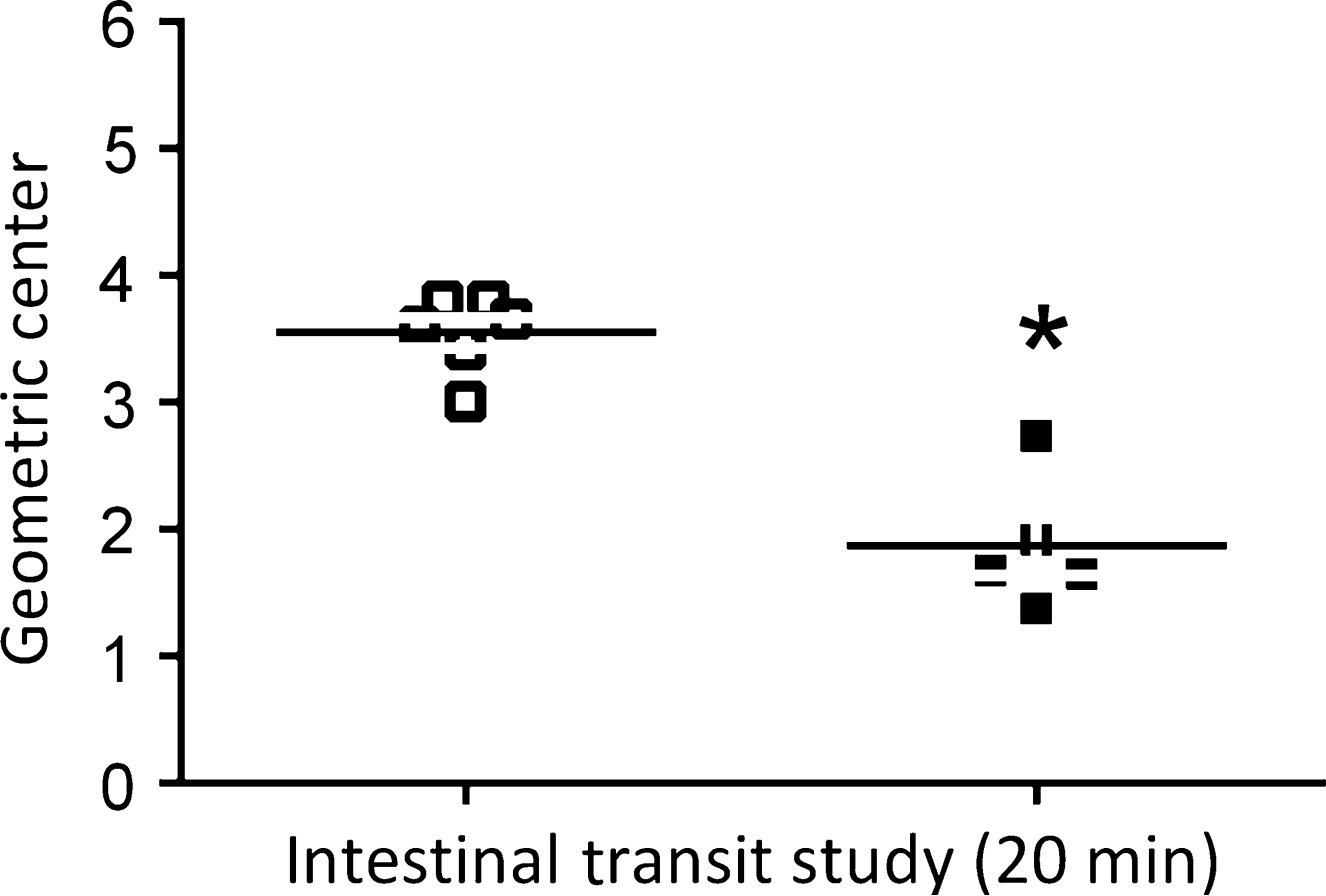
Effect of 5/6 partial nephrectomy (PNX) on dye marker progression through the small intestine in awake rats studied 20 min postprandially. The figure shows the geometric center of mass distribution from the stomach and proximal duodenum (*segment 1*) to the five consecutive small intestine segments (*segment 6*) in awake rats previously subjected (7 days earlier) to PNX (▪) or sham operation (control, □). The horizontal bars are medians of the geometric center of mass. *, *P *<* *0.05 versus control rats after unpaired Student's *t*-test.

The inhibitory effect of PNX on gut motility surpasses the stomach. Figure6 shows that PNX also inhibited the small intestine transit. By evaluating dye marker progression throughout the gastrointestinal tract 20 min after administration of test meal directly into the duodenum, one can see that the center of mass gradually advanced through the gut in sham-operated rats (3.3 [3.0–3.6]) while its respective index remained behind (*P *<* *0.05) in PNX rats (2.9 [2.7–3.1]).

**Figure 7:**
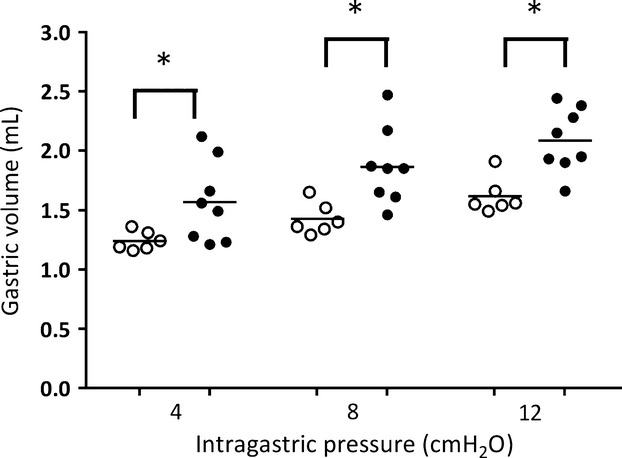
Effects of 5/6 nephrectomy (PNX, •) on gastric compliance in anesthetized rats. The rats were previously subjected 7 days earlier or not (control, ○) to 5/6 nephrectomy. After anesthesia, a balloon catheter was inserted via the fistula at the fundus of the stomach, which was coupled to a plethysmometer to continuously determine gastric volume. Each subgroup consisted of six to nine rats. The data are expressed as mean ± SEM. *, *P *<* *0.05 versus control after unpaired Student's *t*-test.

Figure7 shows that PNX also inhibited the gastric tonus in anesthetized rats. Compared with the respective values in sham-operated (control) rats, stepwise distension of the stomach with intraluminal pressure of 4.0, 8.0, and 12.0 cmH_2_O led to higher (*P *<* *0.01, Student's *t*-test) gastric volumes in the PNX group (1.3 ± 0.1 vs. 2.2 ± 0.3 mL, 1.5 ± 0.1 vs. 2.3 ± 0.3 mL, and 1.7 ± 0.1 vs. 2.4 ± 0.3 mL, respectively).

Table1 shows the biochemical and hemodynamic profiles in sham-operated and PNX rats. Compared with the respective values in the control group, PNX increased (*P* < 0.05) the plasma urea and creatinine values (45.8 ± 3.1 vs. 69.3 ± 2.5 mg dL^−1^ and 0.64 ± 0.05 vs. 0.96 ± 0.07 mg dL^−1^, respectively) as well as the plasma sodium values (138.0 ± 1.7 vs. 141.1 ± 1.5 mEq L^−1^).

## Discussion

This study confirms and extends our previous work (Silva et al. 2002), showing that PNX induced a marked delay both in the GE (28.8-77.1%) and small intestine transit of a liquid test meal in awake rats while enhancing the gastric tonus in anesthetized rats. This phenomenon appears to be elicited by BV imbalance as evidenced by the strong correlation (*r*^2* *^= 0.86) found between hypervolemia and the GE delay. Moreover, the increased gastric retention was prevented by cellular dehydration. The subtotal nephrectomy-induced GE delay appeared to involve cholinergic pathway because it was prevented by either bilateral subdiaphragmatic vagotomy or atropine pretreatment.

Mammals have a large reserve of renal function. We found no conspicuous morphological features in the remnant kidney parenchyma in PNX rats (data not shown). As previously reported (Fleck et al. 2006), the biochemical and hemodynamic changes, with the exception of hypervolemia exhibited by rats 1 week after substantial loss (5/6) of renal parenchyma, were largely unremarkable.

ESRD patients present several changes in gut morphology and physiology, including a decrease in macromolecule permeability and increase in fluid and electrolyte secretion (Hatch et al. 1999; Schoonjans et al. 2002). Clinical reports show heterogeneous data because of large differences in the anthropometric features of patients and the nature of chronic renal failure (CRF). Thus, animal models of ESRD have been designed. Aged, spontaneously hypertensive rats were considered by Zhou and Frohlich (2003) an ideal experimental model for naturally developing ESRD, albeit with an exceedingly high cost to maintain them until 73 weeks of age. A relatively easier technique is chemical nephrectomy, but nephrotoxic drugs have undesirable side effects, such as cardiotoxicity (Liu et al. 2003). The remnant kidney rat model mimics the progressive nephron loss that occurs in human CRF. The reduction of renal mass is usually obtained by a two-stage surgical procedure: partial exeresis of one kidney and total removal of the contralateral kidney. Partial nephrectomy is obtained by resecting the upper and lower poles of the kidney, electrocoagulating the renal cortex, or performing arterial ligation. The resection of both poles of one kidney combined with exeresis of the other kidney is the most commonly used PNX rat model.

ESRD patients present high rates of gastric acid secretion (Goldstein et al. 1967; Gold et al. 1980). The present GE assessment was performed using a dye dilution technique, and the analysis of actual gastric retention could be biased because phenol red is pH-sensitive. However, we previously found that bilateral nephrectomy-induced GE delay was unaltered by omeprazole-induced inhibition of gastric acid secretion (Silva et al. 2002). Thus, the present phenomenon appears to be related to changes in gut motility rather than to eventual hyperchlorhydria.

In rats subjected to chronic uremia, the GE of liquid is not significantly impaired (Raybould et al. 1994). The discordance with the present results may be explained by the strikingly different temporal patterns between these protocols. Chronic uremia is characterized by important morphological and functional abnormalities of the autonomic nervous system that interfere with gut motor behavior, whereas acute azotemia possibly does not allow time for such abnormalities.

Scarce knowledge is available in the biomedical literature about the mechanisms that underlie gastric dysmotility after renal failure. One of several nitrogen compounds that accumulate in the ECF may alter the contractility of gut smooth muscle cells (Lin et al. 1997). In the present work, PNX rats studied one week after the loss of 5/6 renal parenchyma showed higher values of blood urea and creatinine in comparison with the control group (Table1). However, we previously showed that double infusion of urea and creatinine to acutely elevate their blood values at levels similar to those obtained after bilateral nephrectomy did not change the GE rate in awake rats with normal kidney function (Silva et al. 2002).

Our hypothesis is that PNX elicits hemodynamic imbalance, which then alters gut motor behavior. It is supported by a strong correlation (*r*^2* *^= 0.86) between the hypervolemic condition and the increase in gastric fractional dye recovery in PNX rats (Fig.3), whereas cellular dehydration triggered by a subcutaneous injection of polyethylene glycol prevented the PNX-induced GE delay (Fig.4). Moreover, the consumption of a 1% saline diet instead of ORS in PNX rats for 3 postoperative days increased BV and turned statistically significant the GE delay (data not shown). We also found that acute hypervolemia caused by saline or blood infusion increased the resistance offered by the gastroduodenal segment to the flow of liquid in anesthetized rats while delaying the GE of liquid meals in awake rats (Graça et al. 2000, 2002). Functional coupling between the cardiovascular and gastrointestinal systems with regard to BV homeostasis is further strengthened by our previous finding that mechanical distension of the heart by a balloon increases the renal excretion of sodium and water and elicits the GE delay of a liquid test meal in awake rats (Palheta et al. 2013).

In mammals, adequate cardiovascular homeostasis involves a closed circuit that includes afferent sensors located throughout the body that monitor changes in blood pressure, volume status, and the biochemical *milieu* and the central nervous system where these hormones and impulses are integrated via sympathetic and parasympathetic reflexes to peripheral organs, including the heart, vasculature, and kidneys (Antunes-Rodrigues et al. 2004).

In this work, bilateral subdiaphragmatic vagotomy and atropine pretreatment obviated the PNX-induced GE delay, indicating the involvement of descending cholinergic vagal pathways in the present phenomenon. It is further supported by our previous finding that identical parasympathetic denervation also prevented the GE delay caused by saline infusion (Graça et al. 1997). Electrical, mechanical, and chemical cardiac stimuli also increase the afferent trafficking of vagal heart receptors, which elicit vomiting via the vagal reflex and gastric relaxation in cats (Abrahamsson and Thorén 1973). The fact that sympathetic deafferentation reverted the PNX-induced GE delay indicates a role of sympathetic pathways in the present PNX-induced GE delay. In contrast, the GE delay elicited by saline infusion does not involve the sympathetic nerves (Graça et al. 1997).

Additionally, hypervolemia releases atrial natriuretic peptide (ANP), oxytocin, prostaglandins, and nitric oxide (NO), which help mammals excrete salt and water excess, thus restoring homeostasis (Palheta et al. 2010). In a previous work, we showed that mechanical stretch of the right atrium elevated ANP blood levels and delayed the GE of a liquid test meal in awake rats (Palheta et al. 2013). Thus, the involvement of a putative humoral pathway in the present phenomenon should also be taken into account.

The GE of liquid meals is reasoned to be the result of the pressure gradient between the proximal stomach and duodenum, promoting the pulsatile transpyloric flow of liquid, especially during the nonocclusive phase of contractions of the gastroduodenal junction (Rao and Schulze-Delrieu 1993). Because PNX rats exhibited lower gastric tonus, this fact could explain the decreased liquid test meal outflow from the stomach to the small intestine, notwithstanding the obvious influence of anesthesia on the neurohumoral control of gut motor function. In addition, one should also ponder the inhibitory influence of both the pylorus and small intestine on the GE rate, collectively known as the “breaking effect.” In this direction, Fu et al. (2011) observed inhibition of small intestine transit in chronically uremic rats. In the present study, PNX also delayed the progression of the test meal injected directly into the duodenum, indicating an increase in resistance offered by the upper small intestine to the transit of liquid. Fu et al. (2011) also showed that the migratory motor complex is inhibited in chronically uremic rats. Altogether, the results suggest that the GE delay of liquid induced by the progressive loss of nephrons may be involved in gut dysmotility complaints (e.g., bloating and dyspepsia) described by ESRD patients (Van Vlem et al. 2001; Strid et al. 2004). In a previous report, we studied 50 male volunteers with ESRD who were undergoing hemodialysis therapy by means of the ^13^C octanoic acid breath test. We observed that overhydrated ESRD patients had higher *t*_1/2_ and *t*_lag_ values (Salles-Junior et al. 2013).

In conclusion, our results indicated that PNX induced a marked delay in the GE and small intestine transit of a liquid test meal in awake rats and enhanced gastric tonus in anesthetized rats. This phenomenon was prevented by cellular dehydration, and the GE delay was dependent on the increase in BV. The PNX-induced GE delay involved cholinergic pathways, which was prevented by bilateral subdiaphragmatic vagotomy and atropine pretreatment.
